# Vaginal-assisted gasless laparoendoscopic single-site radical hysterectomy for early cervical cancer: a retrospective pilot study

**DOI:** 10.1186/s12957-021-02402-3

**Published:** 2021-09-27

**Authors:** Xiaojuan Wang, Junwei Li, Keqin Hua, Yisong Chen

**Affiliations:** Department of Gynecology, Obstetrics and Gynecology Hospital of Fudan University, 128 Shenyang Road, Shanghai, 200090 China

**Keywords:** Early cervical cancer, Radical hysterectomy, Laparoendoscopic single-site surgery, Gasless pneumoperitoneum

## Abstract

**Background:**

Minimally invasive surgery for early cervical cancer is debated. We developed this new vaginal-assisted gasless laparoendoscopic single-site radical hysterectomy for early cervical cancer, and we aimed to evaluate the feasibility and safety of this surgical procedure and observe the early oncologic outcomes.

**Methods:**

From January 2019 to August 2020, patients with early cervical cancer who underwent vaginal-assisted gasless laparoendoscopic single-site radical hysterectomy were studied retrospectively. The clinical characteristics, pathologic outcomes, perioperative outcomes, and follow-up details of the patients were recorded.

**Results:**

Forty-eight patients underwent vaginal-assisted gasless laparoendoscopic single-site radical hysterectomy were included, 14 (29.2%) with stage IB1, 13 (27.1%) with stage IB2, 7 (14.6%) with stage IB3, 10 (20.8%) with stage IIA1, and 4 (8.3%) stage with IA2. The mean age at diagnosis was 50.4 (range 28–72) years old. The mean operative time was 237.3 min (range 162–393), and the mean estimated blood loss was 246.5 ml (range 80–800). No intraoperative complications occurred, and there were no patients who were readmitted. Histological types were distributed as follows: squamous cell carcinoma 72.9%, adenocarcinoma 10.4%, and adenosquamous cell carcinoma 16.7%. There were 2 patients (4.2%) with positive nodes, 20 patients (41.7%) with positive lymphovascular space invasion, and 2 patients (4.2%) with positive parametria. Twenty-eight patients (58.3%) received adjuvant therapy after the operation. With a mean follow-up of 17.7 months (range 6–26), there were no recurrent cases, and 11 patients (22.9%) suffered lower limb lymphoedema.

**Conclusions:**

The vaginal-assisted gasless laparoendoscopic single-site radical hysterectomy might be a feasible technique for early cervical cancer, with promising short-term oncological outcomes and safety. A prospective study with more patients and longer follow-up periods should be performed to further evaluate the safety and oncological outcomes.

## Background

Cervical cancer ranks as the fourth most frequently diagnosed cancer and the fourth leading cause of cancer death in women [1]. For early cervical cancer, surgery offers several advantages compared with radiotherapy, including preservation of vaginal function, shorter duration of treatment, and avoidance of radiation-induced menopause in younger patients [2]. Radical hysterectomy for early-stage cervical cancer (stage IA1 [with lymphovascular space invasion (LVSI)], IA2 to IIA) could be performed via laparotomy or by minimally-invasive surgery (MIS) [3].

Compared with laparotomy for radical hysterectomy, MIS has the following advantages: less blood loss, fewer blood transfusions, faster time to discharge from hospital, and less febrile episodes and wound infections [4, 5]. Laparoscopic and robot-assisted minimally invasive hysterectomy had gained widespread acceptance as a standard treatment for early-stage cervical cancer. However, the Laparoscopic Approach to Cervical Cancer (LACC) trial, a randomized, open-label, noninferiority study comparing minimally invasive radical hysterectomy with open radical hysterectomy, found that patients with MIS had a higher risk of recurrence and death than those treated with open abdominal radical hysterectomy [6]. Since then, MIS for early cervical cancer has been debated.

Several causes were proposed to explain the high risk of recurrence and poor survival in patients undergoing minimally invasive radical hysterectomy, including application of uterine manipulators, the establishment of pneumoperitoneum through carbon dioxide insufflation, the method of intracorporeal colpotomy, and the surgeon’s experience with MIS [6–9]. It is possible that uterine manipulators, which were frequently used for retraction and visualization during minimally invasive hysterectomy, might disseminate tumor cells and increase the propensity for tumor spillage [6, 7]. The way of intracorporeal colpotomy during laparoscopic surgery was considered to increase likely exposure of the tumor to the abdominal cavity and tumor dissemination [8, 10, 11].

In solid tumor models, CO_2_ pneumoperitoneum had no deleterious effect on tumor growth when compared to gasless laparoscopy or midline laparotomy [12]. However, an in vitro study showed that cervical cancer cells stimulated by the CO_2_ pneumoperitoneum environment could increase the ability of proliferation after a short time of inhibition and reduce the ability of invasion, migration, and adhesion [13]. And the clinical retrospective study showed that exposure of cervical cancer to circulating CO_2_ might result in tumor spillage into the peritoneal cavity and higher recurrence [8, 13]. Therefore, we are not sure whether CO_2_ pneumoperitoneum is at high risk of recurrence and poor survival in patients undergoing minimally invasive radical hysterectomy, and we can take gasless pneumoperitoneum as a protective measure.

How to utilize the advantages of MIS and gain the same outcomes as of laparotomy is so important and interesting to explore. Koehler et al. reported vaginal-assisted laparoscopic radical vaginal hysterectomy consisting of three stages: laparoscopic staging, creation of a tumor-adapted vaginal cuff, and laparoscopic radical hysterectomy, and hysterectomy with minimal intraoperative complications and identical oncologic outcomes [14]. Tergas and Park et al. reported laparoendoscopic single-site (LESS) radical hysterectomy was successfully performed for early cervical cancer [15, 16]. Here, we developed a new surgical procedure that combined the advantages of vaginal-assisted laparoscopic and LESS approaches to perform radical hysterectomy for early cervical cancer with abdominal wall suspension.

This surgical procedure was designed to decrease the tumor spillage and tumor cells dissemination possibly with several strategies: (1) gasless laparoscopy with abdominal wall suspension was performed, (2) enfolding cervical lesions with sterile cloth and creating a vaginal cuff to wrap the lesions further, and (3) devising an extrauterine manipulator without entering the uterine cavity. In this study, we aimed to evaluate the feasibility and effect of the technique for early cervical cancer and early oncologic outcomes.

## Methods

Approval to conduct this study was obtained from the Institutional Review Board at Obstetrics and Gynecology Hospital of Fudan University (Number 2019–32). In our hospital, two doctors performed this new surgical procedure. Before conducting this study, 6 patients underwent this procedure successfully. Every participant had a choice between this new procedure and alternative treatments, such as laparotomy, and radiotherapy, and every participant gave written informed consent and agreed to the operation. For this retrospective study, data were analyzed from January 2019 to September 2020 at the Obstetrics and Gynecology Hospital of Fudan University.

The diagnosis of cervical cancer was based on the International Federation of Gynecology and Obstetrics (FIGO) staging system [17]. All patients with early cervical cancer (stage IA2 to IIA1) who underwent vaginal-assisted gasless LESS radical hysterectomy in our institute were included. Clinical information was obtained from medical records, including age, body mass index (BMI), stage, operating time, blood loss, concomitant operation, tumor histology, tumor size, margin status and distance from tumor, LVSI, parametrial involvement, and depth of stromal invasion, length of hospital stay, time to spontaneous voiding of urine, and intra- and postoperative complications. Patients were required to follow-up regularly based on National Comprehensive Cancer Network (NCCN) clinical practice guidelines in oncology: cervical cancer version 1. 2019: every 3–6 months for 2 years, every 6–12 months for 3–5 years, and then annually based on the patient’s risk of disease recurrence.

### Surgical technique

This vaginal-assisted gasless LESS radical hysterectomy combined the advantages of vaginal-assisted laparoscopy radical hysterectomy and LESS [15, 18], consisted of 3 main parts: (1) gasless LESS staging including lymphadenectomy to evaluate nodal status and bilateral salpingectomy or salpingo-oophorectomy, (2) vaginal creation of a tumor-adapted cuff and dissection of vesicocervical and vesicovaginal septum, and (3) gasless LESS radical hysterectomy with extrauterine manipulator. During the procedure, the measures were designed to avoid tumor spillage and tumor cells dissemination, including gasless pneumoperitoneum, enfolding cervical lesions with sterile cloth, creation of a tumor-adapted vaginal cuff, and extrauterine manipulator.

**Fig. 1:**
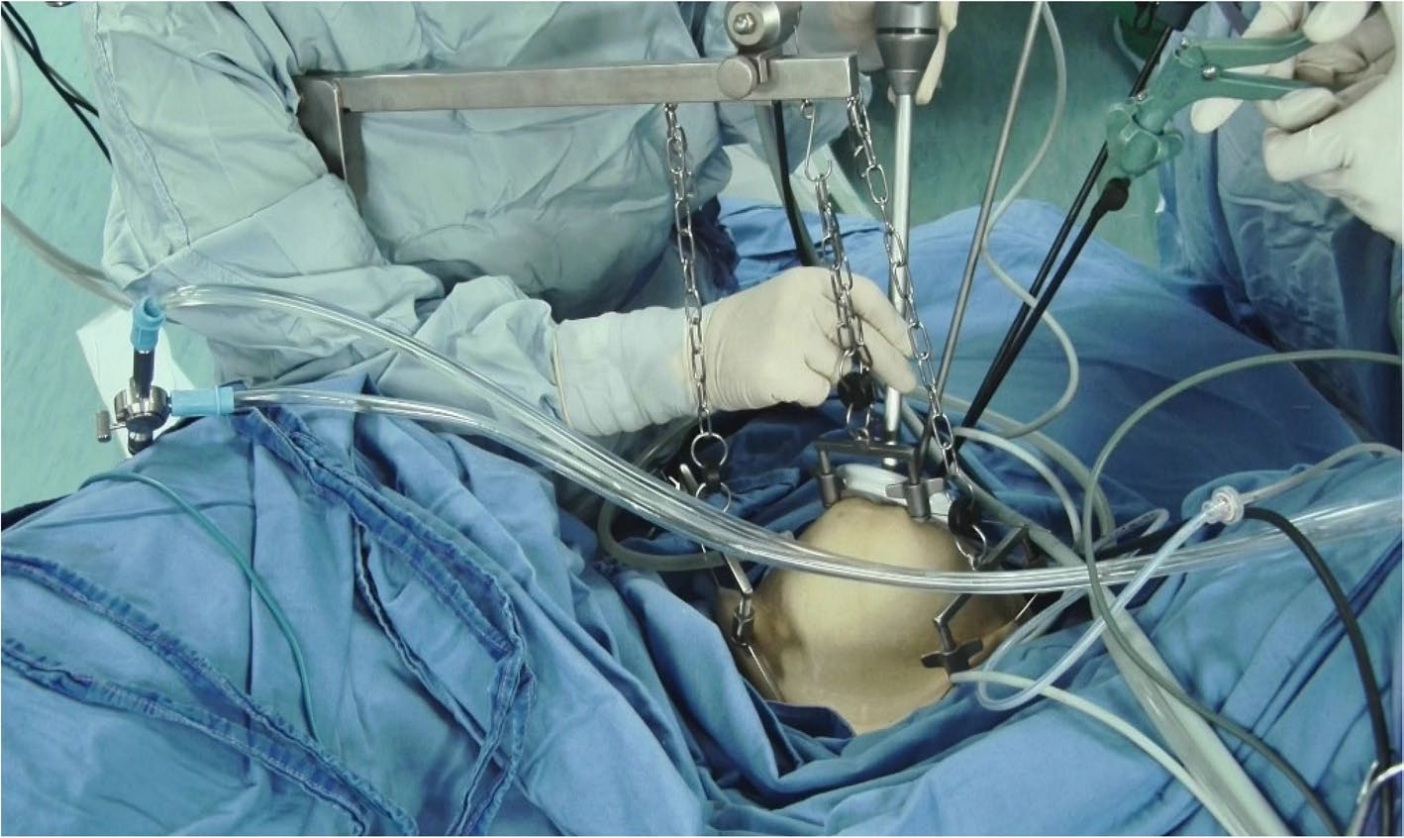
Gasless pneumoperitoneum was built with abdominal wall suspension

For gasless LESS, the patient was placed in a deep Trendelenburg position (30°) with straight legs. We suspended the anterior abdominal wall from one figure below umbilicus, McBurney point, and anti- McBurney point (Fig. 1). A 2-cm vertical incision was made in the umbilicus and abdominal cavity was entered. A small Alexis wound retractor (Applied Medical Systems, Rancho Santa Margarita, CA, USA) was used to provide a smooth and easy surface for the entry of the laparoscope and the laparoscopic instruments. Bilateral salpingectomy or salpingo-oophorectomy and pelvic lymphadenectomy were performed on traditional principles through transumbilical LESS.

**Fig. 2:**
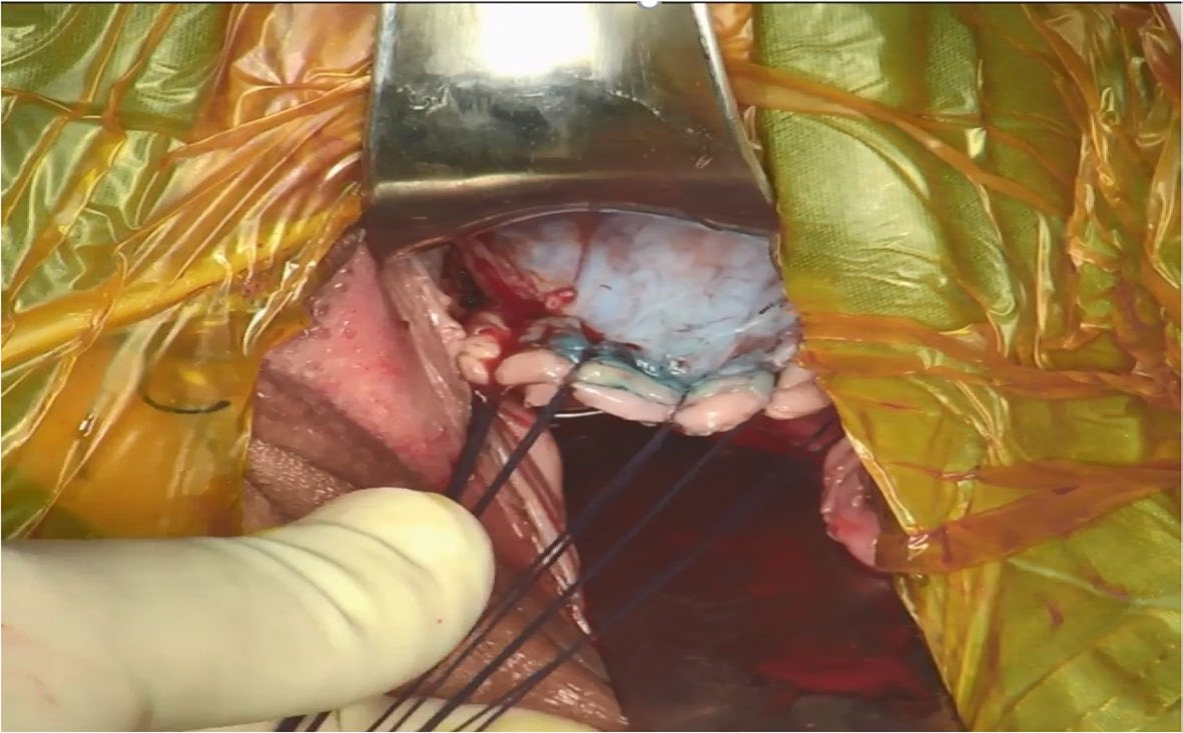
The tumor-adapted vaginal cuff was created

The aim of the vaginal part was to create a tumor-adapted cuff and to open vesicovaginal and rectovaginal spaces. After applying colored iodine to the vaginal wall, sterile cloth was used to enfold cervical lesions. Then, adequate length of the vagina was grasped based on the tumor size. After diluted solution of methylene blue was injected into the vaginal mucosa, vesicovaginal and rectovaginal spaces were separated. The vaginal cuff was closed with six interrupted sutures and then knotted. The tumor-adapted vaginal cuff was created (Fig. 2).

**Fig. 3:**
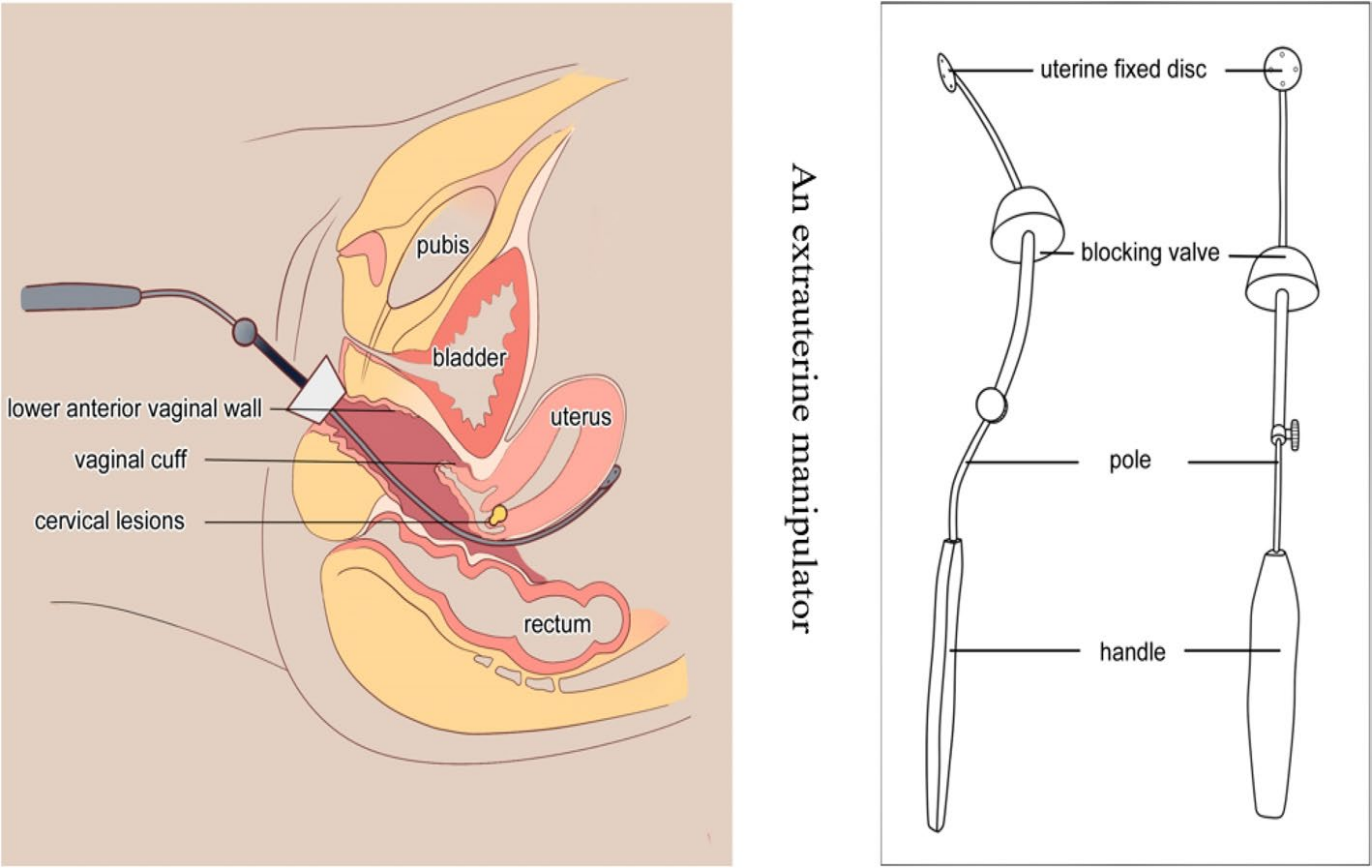
The diagram of extrauterine manipulator

**Fig. 4:**
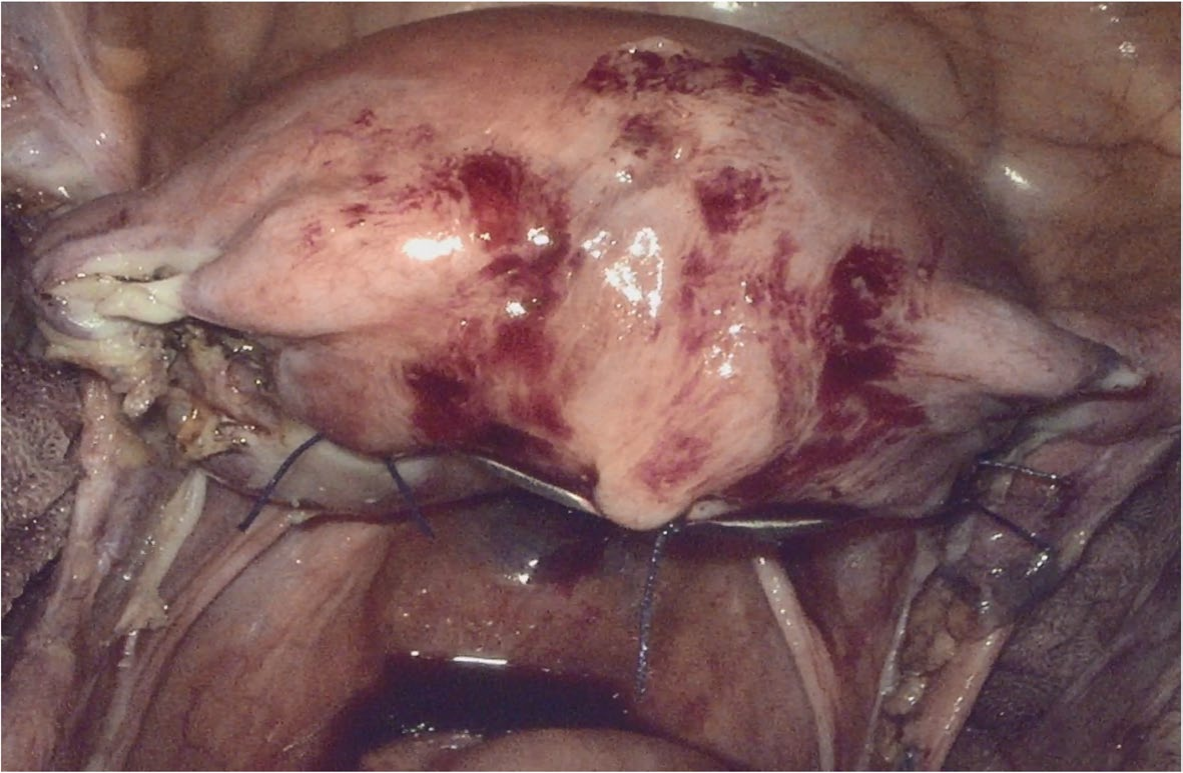
The extrauterine manipulator was placed into the pelvis and fixed

We invented the special manipulator, consisting of a handle, pole, blocking valve, and uterine fixed disc (Fig. 3). The blocking valve was made of silica gel, which was so soft that did not cause crush injuries to the vulva. The uterine fixed disc was round and blunt, with a diameter of 1.2 cm, and there were four eyelets at 3, 9, and 12 o’clock. This extrauterine manipulator was placed through the rectovaginal space, and then rectovaginal space was opened under direct view. The extrauterine manipulator was placed into the pelvis and the uterine fixed disc was placed behind the posterior uterine wall and fixed with sutures (Fig. 4). LESS radical parametrial resection was performed.

We left the urinary catheter in place until the patient was able to empty her bladder spontaneously with residual volume < 100 ml. All patients received low-molecular-weight heparin after operation. Prophylactic antibiotics were given for 48 h after the operation. Subjective symptoms and physical exams were utilized to diagnose lower limb lymphedema. Limb volume was utilized as a metric for measuring lymphedema as a quantitative adjunct to swelling noted by the patient or on physical exam, which relied on a normal limb as an internal standard, or baseline measurements for comparison. If the patient was diagnosed with lower limb lymphedema, color ultrasound was given to exclude venous thrombosis.

### Statistical analysis

Descriptive statistics were expressed in terms of quantitative value as mean standard deviation (SD) or median and range and percentage. Linear regression was conducted to assess the sign of the slope of the regression for the learning curve. Independent *t* tests were used to compare the continuous variables. Statistical analysis was performed using SPSS software (SPSS version 22.0; SPSS Inc., Chicago, IL, USA). Statistical significance was set at *p* < 0.05 for all tests.

## Results

During the study period, a total of 48 patients with early cervical cancer underwent vaginal-assisted gasless LESS radical hysterectomy, and 15 patients refused this new procedure and chose alternative treatment such as laparotomy, traditional laparoscopy, or radiotherapy. The characteristics of the patients included are shown in Table 1. In addition to radical hysterectomy and pelvic lymph node dissection, 41 patients (85.4%) underwent salpingo-oophorectomy, 7 patients (14.6%) underwent ovarian suspension and vaginal extension, 2 patients (4.2%) underwent para-aortic lymph node dissection, and 4 patients were given preventive ureteral stent placement. There was no intraoperative complication occurring. The perioperative details are seen in Table 2.

**Table 1:** Patient characteristics of 48 patients

Characteristic	Value
Age years, mean (range)	50.4 (28–72)
BMI, kg/m^2^, mean (range)	23.2 (17.0–31.6)
Parity, mean (range)	1.60 (1–6)
Medical comorbidities, number (%)	16 (33.3%)
Hypertension, number (%)	11 (22.9%)
Diabetes mellitus, number (%)	5 (10.4%)
Previous pelvic surgery, number (%)	11 (22.9%)
Stage, number (%)	
IA2	4 (8.3%)
IB1	14 (29.2%)
IB2	13 (27.1%)
IB3	7 (14.6%)
IIA1	10 (20.8%)
Pathology details	
Squamous cell carcinoma, number (%)	35 (72.9%)
Adenocarcinoma, number (%)	5 (10.4%)
Adenosquamous cell carcinoma, number (%)	8 (16.7%)

*BMI* body mass index

**Table 2:** Perioperative details of 48 patients

Characteristics	Values
Operation time, minutes mean (range)	237.2 (162–393)
Estimated blood loss, milliliters mean (range)	246.5 (80–800)
Blood transfusion, number (%)	2 (4.2%)
Salpingo-oophorectomy	41 (85.4%)
Ovarian suspension and vaginal extension	7 (14.6%)
Para-aortic lymph node dissection	2 (4.2%)
Preventive ureteral stent placement	4 (8.3%)
Intraoperative complications	0
VAS score after operation, mean (range)	3.1 (1–5)
Length of hospital stay, nights mean (range)	7.2 (3.5–23)
Fever	6 (12.5%)
Readmission	0

The mean length of anterior vaginal wall was 2.9 cm (range 2–4), the mean length of posterior vaginal wall was 3.4 cm (range 2.5–5), and the mean length of parametria was 2.6 cm (range 2.2–3). There were 2 patients (4.2%) who had positive nodes. There were 20 patients (41.7%) who had positive LVSI, and 2 patients (4.2%) had positive parametria. Twenty-eight patients (58.3%) received adjuvant therapy after the operation. The other postoperative histological results are shown in Table 3.

**Table 3:** Postoperative histological results

Characteristics	Value
The length of anterior vaginal wall, cm mean (range)	2.9 (2–4)
The length of posterior vaginal wall, cm mean (range)	3.4 (2.5–5)
The length of parametria, cm mean (range)	2.6 (2.2–3)
Positive nodes, number (%)	2 (4.2%)
LVSI ( +), number (%)	20 (41.7%)
Parametria ( +), number (%)	2 (4.2%)
Stromal invasion	
Deep 1/3, number (%)	21 (43.7%)
Superficial 1/3, number (%)	6 (12.5%)
Tumor size	
< 2 cm, number (%)	20 (41.7%)
2–4 cm, number (%)	20 (41.7%)
> 4 cm, number (%)	7 (14.6%)
Adjuvant therapy, number (%)	28 (58.3%)

*cm* centimeter

**Fig. 5:**
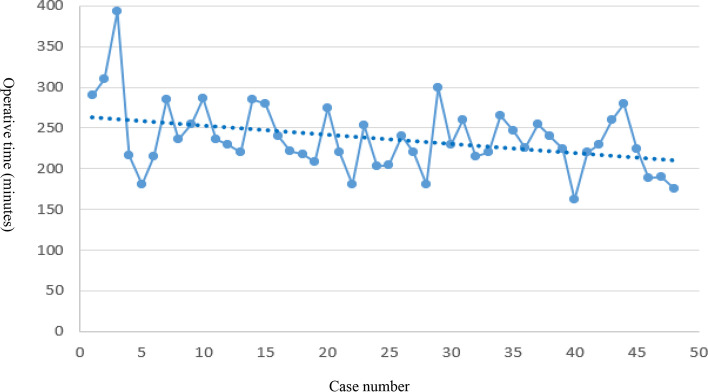
Learning curve

We compared the first 24 procedures and the sequential 24 procedures and found the mean operative time greatly decreased from 247.2 min (± 47.3) in the first 24 patients to 213.5 min (± 26.2) in the following 24 patients (*p* = 0.004). The length of hospital stay decreased from 8.25 nights (± 2.9) in the first 24 patients to 5.5 nights (± 1.8) in the sequential 24 patients (*p* < 0.001). There was no difference in estimated blood loss or hemoglobin (Hb) drop in the first 24 patients and the sequential 24 patients. The above was seen in Table 4 and Fig. 5.

**Table 4:** The comparison between the first 24 procedures and the sequential 24 procedures

	Patients 1–24	Patients 25–48	*P* value
Operation time, minutes, mean (SD)	247.2 ± 47.3	213.5 ± 26.2	0.004
Estimated blood loss, milliliters, mean (SD)	250.0 ± 148.2	242.9 ± 112.8	0.853
Length of hospital stay, nights mean (SD)	8.2 ± 2.9	5.5 ± 1.8	< 0.001
Hb drop (g/dl), mean (SD)	2.0 ± 1.5	1.8 ± 1.0	0.628

*SD* standard deviation

There were no loss-to-follow-ups. After a mean follow-up of 17.7 months (range 6–26), there were no recurrent cases. The mean time of indwelling urinary catheter removal with residual urine < 100 ml was 33.5 days (range 18–90). During follow-up, 11 patients (22.9%) suffered lower limb lymphoedema, and no patients experienced venous thrombosis. The outcomes of follow-up are seen in Table 5.

**Table 5:** The outcomes of follow-up

Variables	Value
The length of follow-up, months, mean (range)	17.7 (6–26)
Time to urinary catheter removal, days, mean (range)	33.5 (18–90)
Lower limb lymphoedema, number (%)	11 (22.9%)
Venous thrombosis, number (%)	0
Recurrence, number (%)	0

## Discussion

Radical hysterectomy is the operative standard procedure for early cervical cancer. There is a broad spectrum of open, total laparoscopic, laparoscopic-assisted vaginal, robotic, and LESS techniques for cervical cancer [18–21]. However, a retrospective study and a multicenter prospective randomized controlled trial published in 2018 showed that the disease-free survival (DFS) and overall survival (OS) of patients with early cervical cancer who underwent MIS were significantly lower [6, 7], and MIS radical hysterectomy is debated. Some studies showed that MIS radical hysterectomy for cervical cancer did not confer worse oncologic outcomes in a single-center; the 5-year DFS rates were 87% in the MIS group and 86.6% in the laparotomy group (*p* = 0.15) [22]; and MIS therapy for cervical cancer improved DFS [23]. Some studies showed that laparotomy resulted in better OS and DFS than MIS among patients with stage IB cervical cancer (94.1 vs. 87.5%, 98.1 vs. 92.3%) [24]. The surgical approach might impact on the oncologic outcomes in women undergoing radical hysterectomy for cervical cancer [25]. In our study, we found that vaginal-assisted gasless LESS radical hysterectomy was associated with fewer perioperative complications and promising oncological outcomes with a mean follow-up of 17.7 months.

We developed this new surgical procedure for radical hysterectomy to improve the oncological outcomes of MIS for cervical cancer. The vaginal cuff enfolded the cervical lesion, which theoretically decreased the tumor spillage and tumor cells dissemination to the greatest extent. The extrauterine manipulator was utilized, which was convenient to perform LESS. It is reported that the continuously perfusing and flowing carbon dioxide in the abdominopelvic cavity could lead to spread of the detached tumor cells [8, 9, 13]. Regardless of whether the CO_2_ pneumoperitoneum environment increased tumor-cell growth or spread, our gasless laparoscopy was similar to laparotomy, which may be a protective measure. This new surgical procedure for radical hysterectomy should result in better DFS and OS. During the mean 17.7 months of follow-up, we found that there were no recurrent cases and that there were no deaths after surgery, which was significantly less than that in previously reported publications [14]. Based on the pilot outcomes, this vaginal-assisted LESS radical hysterectomy for early cervical cancer might be a feasible procedure. However, a prospective random controlled trial study with longer-term follow-up is needed.

Even though the mean operative time in our study was higher than the mean operative time of previous publications [26, 27], from the learning curve, we could see that the mean operative time should be shortened in the future. The estimated blood loss and hospital stay were less than the estimated blood loss and hospital stay in a previous publication [28, 29]. No intraoperative complications occurred in our study. Preventive ureteral stent placement was performed for 4 cases (8.3%) and later removed 2 months after the operation successfully without ureteral injury, which was based on the surgeon’s experience. The rate of lower extremity lymphedema occurring (22.9%) after radical hysterectomy seemed higher, which was kept in accordance with one randomized controlled trial in our hospital (23.9%) [30]. However, the reported incidence of lower extremity lymphedema fluctuates dramatically, with rates ranging from 1.2 to 37.8% [31, 32]. These variations in incidence among publications might be attributed to a lack of criteria for evaluating lymphedema. Different definitions, including an objective judgment by physicians, subjective complaints from patients (“symptomatic” lymphedema), or a mixture of both, have been utilized in previous publications. We used subjective symptoms and limb volume to identify the lower extremity lymphedema. After physiotherapy intervention, the lower extremity lymphedema rate in our institution was decreased to 13.6% [30]. How to decrease and prevent the lower extremity lymphedema should be managed in the future.

The urinary catheter indwelling time in our study was significantly longer than the urinary catheter indwelling time in publications [33], which was due to our delayed indwelling urinary catheter removal. We often regularly removed the urinary catheter one month after operation. If the postvoid residual test was higher than 100 ml, the urinary catheter was indwelled again and removed 1 month later. Nerve-sparing radical hysterectomy was reported to preserve voiding function and bladder sensation at 1 year and did not appear to compromise oncological outcome [34]. Some cases in our study were nerve sparing, and we should try to remove the urinary catheter earlier in the future.

To our knowledge, this was the first article to describe the surgical experience of vaginal-assisted gasless LESS radical hysterectomy for early cervical cancer. We also recognized some limitations; the limited sample size and short follow-up time might not reflect the true incidence of surgical complications and oncological outcomes; this was an observational study with no comparative arm. Next, a prospective randomized controlled trial study should be performed to further evaluate this new surgical procedure for cervical cancer.

## Conclusions

Our pilot experiences suggested that vaginal-assisted gasless LESS radical hysterectomy might be a feasible technique for early cervical cancer, with promising short-term oncological outcomes and safety; meanwhile, this procedure had the advantages of all minimally invasive approaches, such as fast recovery and esthetic advantages.

## Data Availability

The datasets used and analyzed during the current study are available from the corresponding author on reasonable request.

## Abbreviations

LESS: Laparoendoscopic single-site
LVSI: Lymphovascular space invasion
MIS: Minimally-invasive surgery
LACC: The Laparoscopic Approach to Cervical Cancer
FIGO: The International Federation of Gynecology and Obstetrics
BMI: Body mass index
Hb: Hemoglobin
SD: Standard deviation
DFS: Disease-free survival
OS: Overall survival
